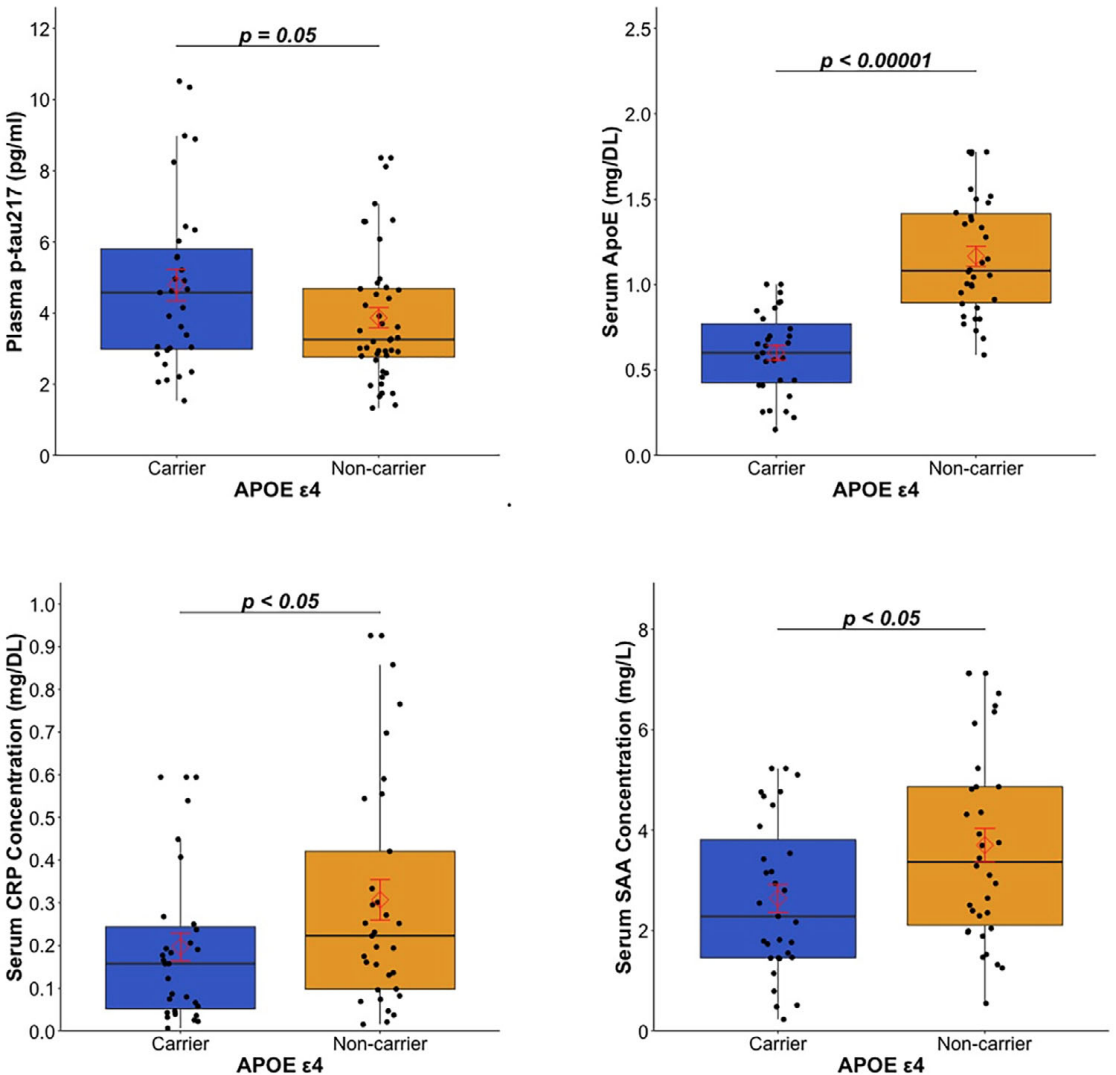# Effects of APOE ε4 on novel blood biomarkers of amyloid‐β pathology and inflammation in midlife adults at risk of Alzheimer’s disease

**DOI:** 10.1002/alz.095617

**Published:** 2025-01-09

**Authors:** Emily Bechke, Kyoung Shin Park, Laurie Wideman, Travis Anderson, Samantha J Goldenstein, Alexis B Slutsky‐Ganesh, Brittany D Armstrong, Jennifer L Etnier

**Affiliations:** ^1^ University of North Carolina at Greensboro, Greensboro, NC USA; ^2^ United States Olympic and Paralympic Committee, Colorado Springs, CO USA; ^3^ Emory University, Atlanta, GA USA

## Abstract

**Background:**

In APOE ε4 allele carriers at increased risk for Alzheimer’s disease (AD), midlife (age 40‐65 years) is a critical window when amyloid‐β (Aβ) pathology could progress without overt cognitive decline, but AD‐related biomarkers may be present. Blood measures of phosphorylated tau at Thr217 (p‐tau217) indicate aggregated Aβ pathology in the preclinical stage of AD, whereas ApoE protein levels are associated with the clearance of Aβ. C‐reactive protein (CRP) and serum amyloid A (SAA) are acute phase reactants within the inflammatory pathway, but SAA is also related to ApoE levels and Aβ clearance. This study aims to investigate the cross‐sectional effects of APOE ε4 carriage on blood biomarkers of Aβ pathology (p‐tau217 and ApoE) and inflammation (CRP and SAA) in midlife adults at risk of AD.

**Method:**

Baseline data from an ongoing clinical trial (NIH R01AG058919) were analyzed. Participants (n = 75, age = 57±6.15 years) were cognitively normal, middle‐aged adults with a family history of AD. APOE ε4 carriage was determined from passive drool saliva samples. Fasting samples were collected, centrifuged at 2,000 g for 12 min at 4°C and 0.5 ml of serum/plasma was aliquoted into tubes and frozen at 80°C. All biomarkers were measured using electro‐chemiluminescence immunoassays. After winsorizing at z = 2‐2.5, analyses of covariance compared the biomarkers between APOE ε4 carriers (n = 31) and non‐carriers (n = 44) with age, sex, education, and BMI as covariates.

**Result:**

After covariate adjustments, APOE ε4 carriage was associated with all serum/plasma biomarkers. APOE ε4 carriers had higher p‐tau217 (p = 0.05), lower ApoE (p<0.0001), lower CRP (p<0.05), and lower SAA (p<0.05) compared with non‐carriers.

**Conclusion:**

Blood biomarkers of greater Aβ aggregation and less systemic inflammation were found in cognitively normal, APOE ε4 carriers in midlife. This data suggests that even in midlife adults at risk for AD due to APOE ε4 carriage, blood biomarkers provide crucial information about early changes in Aβ pathology and provide a potential conduit for maximizing brain health by pinpointing optimal timing for interventions that mitigate risk of progression to AD. The content of this abstract is solely the responsibility of the authors and does not necessarily represent the official views of the NIH.